# Regeneration of Damaged Tendon-Bone Junctions (Entheses)—TAK1 as a Potential Node Factor

**DOI:** 10.3390/ijms21155177

**Published:** 2020-07-22

**Authors:** Nina Friese, Mattis Benno Gierschner, Patrik Schadzek, Yvonne Roger, Andrea Hoffmann

**Affiliations:** 1 Department of Orthopedic Surgery, Graded Implants and Regenerative Strategies, OE 8893, Laboratory for Biomechanics and Biomaterials, Hannover Medical School (MHH), 30625 Hannover, Germany; Friese.Nina@mh-hannover.de (N.F.); Gierschner.Mattis@mh-hannover.de (M.B.G.); Schadzek.Patrik@mh-hannover.de (P.S.); Roger.Yvonne@mh-hannover.de (Y.R.); 2 Lower Saxony Centre for Biomedical Engineering, Implant Research and Development (NIFE), 30625 Hannover, Germany

**Keywords:** enthesis organ, embryonic development, adult, regeneration, inflammation, immune system, TAK1

## Abstract

Musculoskeletal dysfunctions are highly prevalent due to increasing life expectancy. Consequently, novel solutions to optimize treatment of patients are required. The current major research focus is to develop innovative concepts for single tissues. However, interest is also emerging to generate applications for tissue transitions where highly divergent properties need to work together, as in bone-cartilage or bone-tendon transitions. Finding medical solutions for dysfunctions of such tissue transitions presents an added challenge, both in research and in clinics. This review aims to provide an overview of the anatomical structure of healthy adult entheses and their development during embryogenesis. Subsequently, important scientific progress in restoration of damaged entheses is presented. With respect to enthesis dysfunction, the review further focuses on inflammation. Although molecular, cellular and tissue mechanisms during inflammation are well understood, tissue regeneration in context of inflammation still presents an unmet clinical need and goes along with unresolved biological questions. Furthermore, this review gives particular attention to the potential role of a signaling mediator protein, transforming growth factor beta-activated kinase-1 (TAK1), which is at the node of regenerative and inflammatory signaling and is one example for a less regarded aspect and potential important link between tissue regeneration and inflammation.

## 1. Introduction

The functionality of the musculoskeletal system is based on force transmission between muscles and bones, which are connected by tendons. Due to the differential mechanical properties of elastic soft tendons and stiff hard bones, the attachment unit, called enthesis, needs to fulfil high compositional, organizational and mechanical requirements. These demanding properties make entheses an extraordinary and interesting tissue interface. Because of these interesting facts, several reviews have already been written about enthesis before [1,2,3]. In the first part of the present review, we give a short overview on the structure, development and physiology of this special tissue. We continue by summarizing tissue engineering strategies. The major part of the review is devoted to pinpointing the potential role of TAK1 as a future therapeutic target.

### 1.1. Two Types of Entheses

**Figure 1:**
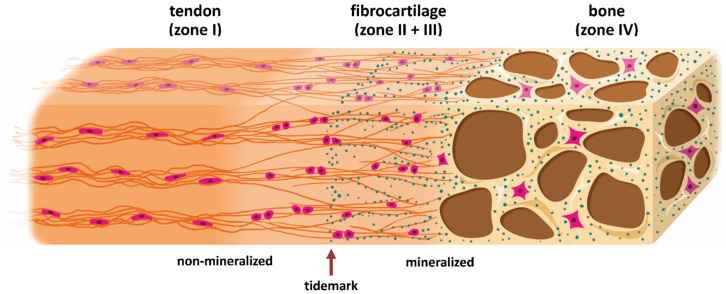
Schematic representation of a direct enthesis with four interwoven zones. Histologically, the basic scaffold is based on a largely oriented fiber course of collagens and a corresponding orientation of the cell axes in the tendon zone (I), while cartilage and bone zones have less or no such orientation of the fibers. Major differences in the composition result from different cell types, molecules of the extracellular matrix and mineral content. The tendon zone contains elongated, fibroblast-like cells, called tenocytes, embedded in an extracellular matrix of predominantly collagen type I, proteoglycans such as aggrecan and fibromodulin as well as glycoproteins like lubricin, elastin and tenascin C [7]. The fibrous cartilage zone II contains rounded chondrocytes in an extracellular matrix which transitions from collagen type I to collagen type II and collagen type III, together with aggrecan as a major non-collagenous protein. Collagen type II is also found in the mineralized fibrous cartilage zone (III), forming a mesh which anchors tendons to bone [7]. The transition between zone II and III, from non-mineralized to mineralized fibrocartilage, is marked by dense calcification, which is called tidemark. In addition, mineralization based on calcium phosphate begins (small turquoise-colored dots). The mineralization is pronounced in the bone zone (zone IV) and contains also crystals (larger turquoise-colored dots). Individual collagen type I fibers are not shown here. Osteoblasts, osteocytes and osteoclasts are the major cell types within bone. The highly vascularized bone provides nutrients and, if applicable, cells to the avascular enthesis [12]. This highly specialized structure – about 0.5 mm in length [15] – results in different mechanical properties which guarantee the enormous stability and thus the functionality of such a transition in vivo.

Tendon-bone transitions occur in two forms in the body—fibrous and fibrocartilaginous [2,3]. In fibrous entheses the dense collagen fibers of the tendon directly enter the bone. Fibrous entheses are present in tendons and ligaments which attach to the metaphyses or diaphyses of long bones [4]. In contrast, fibrocartilaginous entheses as they are present in epiphyses and apophyses [4] represent a complex gradient of unmineralized to mineralized tissues which are conventionally divided into four zones [5,6]. Figure 1 provides a schematic illustration of a fibrocartilaginous enthesis. The first zone (region I in Figure 1) is the tendon (or ligament in case of bone-to-bone junctions) which consists of fibroblast-like cells, called tenocytes. Highly aligned collagen type I fibers are interspersed in a non-collagenous extracellular matrix consisting of proteoglycans and glycoproteins as major components [7]. The following zone (II) is made up of uncalcified fibrocartilage with fibrochondrocytes in a network-like organized collagen type II and III fibers as well as proteoglycan aggrecan as major constituents. The third zone (III) is calcified fibrocartilage with collagen type II and X stiffened by mineral deposits and aggrecan. The transition area ends in bone as fourth zone (IV) and consists of osteoblasts, osteocytes and osteoclasts in a matrix of mineralized collagen type I [8,9,10,11]. The bone at these sites is thin and porous. Blood vessels enter this region from the adjacent bone marrow and thereby provide nutrition to the entheses and allow the removal of metabolic products [12]. In contrast to this, the fibrocartilage area is an avascular zone which decreases the transmission of inflammation and infection from vascularized bone to less vascularized tendon [6].

In the cartilaginous transition zone (regions II and III), the mineral content increases relative to collagen resulting in a gradual increase in stiffness [13,14]. The border between non-mineralized and mineralized fibrocartilage is marked by a calcification front, called tidemark, see also Figure 1. The collagen fibers continue across this tidemark and gradually change from tendon fibers into thinner interface fibers; finally, groups of these fibers enter the bone [15]. The transition zone [6] which measures about 500 µm [15] ensures that tensions are minimized by providing elasticity and stiffness which are required for high mechanical demands within entheses. Important for stress distribution and mechanical stability, the fibers do not form simply a continuous network but may end freely in the interstitial matrix. This is supported by a high density of hydroxyapatite crystals in the mineralized fibrocartilage zone which are not permanently fixed in the matrix but freely move along the fibers [15,16]. Such arrangement results in an enormous enlargement of the interaction areas. The connection between bones and tendons in the body is consequently ideally put into action by the transitional tissue with its characteristic properties which ensures the transmission of force.

Summarizing, the four zones are only compositionally distinct but functionally constitute a continuous system with graded transitions in their properties. Due to the high complexity and interaction of the enthesis with its surrounding tissues, Benjamin & McGonagle (2001) even called it an enthesis organ [17]. Components of the enthesis organ are the enthesis, subtendinous bursae and fibrocartilages. Within this complex, the fibrocartilages protect tendons and bones from compression or abrasion by replacing a synovium [12,15,16].

### 1.2. Development of the Enthesis

The formation of the enthesis begins during the fetal development and continues even postnatally [18]. Chondrocytes of the cartilage start to proliferate and synthesize extracellular matrix until they reach hypertrophy. Thereupon, the cartilage is mineralized by endochondral ossification and collagen fibers and minerals are remodeled by fibrocartilaginous transition. An infiltration of vascular tissue into the mineralized fibrocartilage, which brings bone precursor cells into the enthesis, allows these cells to remodel parts of the mineralized matrix into bone [19,20].

During embryonic development of bones two types of progenitors are involved. Progenitors of the primary cartilage template only express sex-determining region Y-box 9 (SOX9). Another progenitor population is influenced by the opposing expression of gradients of SOX9 and scleraxis (SCX) [21,22]. These progenitors are added to the primary cartilaginous template and form bone eminences which act as anchoring points for tendons in the attachment unit [23]. As it is also expressed in progenitors of primary cartilage, the transcription factor SOX9 is a key molecule in chondrocyte differentiation and continuously active during early and advanced chondrogenesis. SCX is also a transcription factor and pivotally regulates differentiation of tendinous tissues. Knockout experiments revealed that loss of SCX expression results in drastic developmental disorders in the enthesis like changes in cell morphology, fiber organization and enthesis size [21]. As development and differentiation into tenocytes and chondrocytes progresses, the pool of SOX9 and SCX-expressing progenitors gradually decreases.

These developmental changes are driven by several molecular mechanisms, mainly by members of the transforming growth factor-β (TGF-β) family. The TGF-β subfamily has a key role in the formation of the attachment unit since TGF-β signaling controls the specification of bone eminence progenitors and is essential for tendon and cartilage formation [23]. Without functional TGF-β signaling SCX is not expressed and this would lead to a defective development of tendons and ligaments [24]. Besides TGF-β, BMPs are promising factors for tendon healing. One key mediator of tendon-specific signaling during enthesis development is the bone morphogenetic protein-4 (BMP-4). It is co-localized with SCX in bone eminence progenitor cells and is also regulated by SCX. This SCX-driven BMP-4 signaling induces the bone eminence formation at the tendon-cartilage attachment area. Without SCX/BMP-4 signaling the differentiation of these progenitor cells into cartilage is blocked and they remain as SOX9-positive progenitor cells [20,23,24].

Furthermore, the fibrocartilaginous mineralization and maturation of the enthesis is regulated by different molecular signaling cascades. At the tendon-cartilage interface, a population of hedgehog (HH)-responsive cells mediate the development of a mineral gradient shortly after birth. These cells remain in the developing enthesis and populate the region of fibrocartilage whereby just the cells in the non-mineralized parts remain HH-responsive. Chondrocytes in the mineralized fibrocartilage stop expressing factors of the HH-signaling pathway, which leads to a graded expression and therefore to a graded transition between mineralized and unmineralized tissue [21,25,26]. The mineralization process is regulated by a negative feedback loop of Indian hedgehog (IHH) and the parathyroid hormone-related protein (PTHrP) [27]. IHH is expressed by hypertrophic chondrocytes and regulates the rate of their differentiation and proliferation. Additionally, the synthesis of the downstream protein PTHrP is stimulated by IHH. Thus, PTHrP modulates the rate of hypertrophic differentiation and chondrocyte maturation by blocking further expression of IHH [20,28,29]. Both, HH signaling and a proper mechanical load are mandatory for mineralization and maturation of the transition area. Lack of HH signaling leads to decreased fibrocartilage mineralization and consequently results in reduced mechanical properties of the enthesis. On the other hand, the IHH-expressing chondrocytes are modulated by mechanical load. Without muscle forces, especially at later developmental stages, a higher number of chondrocytes stays HH-responsive and does not maturate [25,30,31,32].

## 2. Approaches towards Restoration or Regeneration of a Damaged Enthesis

Tissue damages are caused by infections, toxic and mechanical injuries including overuse, degeneration or combinations of these factors. Furthermore, the steady advancement of civilization also leads to an increase in metabolic diseases like obesity and diabetes, which further increase the risk of orthopedic damage. The natural healing process of damaged tendon-bone junctions is unsatisfying and fails to restore its original functionality [33]. Up to date, the artificial reconstruction of tendon-bone junctions by medical intervention is an unresolved issue. Current surgical restoration methods are suboptimal since enthesis repair by suture of a tendon to a bone via bone anchors does not result in functional restoration but in degenerative and little vascularized tendons, muscle atrophy and lack of integration into the bone [34]. The absence of well-established natural or artificial transplants or implants leads to an immense necessity to promote research and action in this field. A major challenge is the biological, chemical and physical variance in the enthesis, described above. Any replacement material needs to create a connection between these variances. Therefore, the biological aspect is going to become of growing importance in the future [33] – implying the need for solutions to unresolved biological questions.

In line with this argument, cellular and molecular signals are more and more in the center of interest. Above all, the attempt to generate fibrocartilaginous entheses with their four zones and at their anatomically correct location has failed largely so far (see for example, in References [35,36,37]). In these approaches, constructs with zones made of materials with defined properties without continuous gradients were used. On the other hand, materials with gradients of mainly just one single parameter—for example, the mineral content—were applied (e.g., References [38,39]). Unfortunately, not all of these experimental approaches allowed a controlled gradient (e.g., Reference [40]).

Lu et al. (2006) [40] described a three-zone scaffold—zone A made of knitted, degradable poly(lactic-co-glycolic acid) fabric for tendon, zone B made of sintered degradable polymer microparticles for the fibrous cartilage region with a low calcium phosphate content and zone C made of osteo-integrative polymer-ceramic composite microparticles with a high calcium phosphate content for the bone zone [41,42,43]. In the next steps, a tissue engineering approach was used by seeding fibroblasts in zone A, chondrocytes in zone B (if applicable) and osteoblasts in zone C in vitro and subsequently examined in vivo. The subcutaneous (i.e., ectopic) implantation of scaffolds populated with fibroblasts and osteoblasts in nude rats showed that the compression modulus of the biodegradable scaffold decreased in vivo due to degradation, however this effect was compensated by the production of extracellular matrix [42,43]. In the setup with all three cell types, a fibrocartilaginous transition was formed in experimental animals subcutaneously. The compression modulus of the explanted scaffolds initially decreased over time but increased again due to the production of matrix and tissue infiltration [44]. Unfortunately, the behavior of these scaffolds was not tested orthotopically at a real tendon-bone junction in vivo.

The first attempts to produce degradable implants with graded properties were carried out based on retroviruses. Collagen sponges were coated in a gradient with poly-l-lysine, which was generated by using a motorized dip coater and different dipping times. Since poly-l-lysine is a cationic polymer, the poly-l-lysine gradient allowed the formation of a corresponding gradient of retroviruses whose shell was negatively charged. These retroviruses encoded an osteogenic transcription factor, runt-related transcription factor 2 (*RUNX-2*). Finally, skin fibroblasts were applied to test this material in vitro. After transduction with the retrovirus, the cells developed into cells similar to osteoblasts which caused a gradient of cell types corresponding to the original poly-l-lysine gradient. A mineral gradient was developed and could be shown by microcomputed tomography. After subcutaneous implantation of these cell-seeded materials in rats biomechanical tests were carried out, which showed a higher Young’s modulus on the retrovirus-carrying side [45]. Unfortunately, orthotopic in vivo studies and histological examinations, which would verify the formation of tendon-bone transitions, are missing.

In additional studies, the potential for delivering adenovirus or lentivirus encoding green fluorescent protein, beta galactosidase or firefly luciferase from tissue engineering scaffolds using a surface immobilization strategy was assessed. The authors demonstrated that most of the immobilized lentivirus was successfully released from the poly(lactide-co-glycolide) scaffold within 24 h while the adenovirus approach was not further pursued [46]. After subcutaneous implantation, luciferase expression was visualized during a time course of four weeks [46]. In the related field of osteochondral tissue transitions, lentiviruses encoding cDNA for either the chondrogenesis-inducing signaling protein *TGF-β3* or the osteogenesis-inducing protein *BMP-2* were spatially deposited within anatomically-shaped cartilage-derived matrix. In addition, to inhibit aberrant inflammatory processes, lentiviral particles encoding IL-1 receptor antagonist (*IL1RN*) were also immobilized. Subsequent experiments with human mesenchymal stem cells (MSCs) demonstrated successful spatial differentiation into cartilage and bone cells [47].

Another approach was based on the graded mineralization of a scaffold from biodegradable material [39]. Here, fiber mats—produced by electrospinning of poly(lactic-co-glycolic acid) or polycaprolactone—were coated with a solution of calcium phosphate after plasma treatment and/or gelatin coating by means of varied contact times. This coating resulted in graded mechanical properties and a graded colonization with MC3T3-E1 cells, a mouse cell line which was established from calvariae and forms bone cells. Here, in vivo experiments are missing as well [39]. For future applications, it seems important to be able to control the mineralization in relation to the modification of calcium phosphate and to supplement the mechanical and chemical gradients by a “biochemical” gradient (in e.g., growth and differentiation factors).

In this respect, temporally controlled release of biologically active factors can be an additional tool to implement chemical signal gradients and to generate continuous three-dimensional gradients [48,49]. It was demonstrated that growth factors or other molecules could be released for at least 30 days from a precise position within a three-dimensional scaffold structure. For this purpose, microparticles loaded or unloaded with bone morphogenetic protein-2 (BMP-2) were produced [50], layered in different zones and converted into an interlocking but porous structure by sintering. The amount of released BMP-2 could be adjusted by mixing the protein-loaded with unloaded microparticles. Due to the BMP-2 release a local differentiation of bone cells from mesenchymal progenitor cells (murine cell line C2C12) was achieved in vitro [50]. Microparticles from silk fibroin were used to embed BMP-2 and insulin-like growth factor-1 in porous carrier materials and supported the bone and cartilage cell formation from MSCs. Here, joint and opposing concentration gradients of the two factors could be established; however, the incorporation and release of insulin-like growth factor-1 was limited. The temporal release of both factors was different and was followed for 14 days [51]. Another report showed that by embedding poly(lactic-co-glycolic acid) microparticles in carrier material which is made of poly(lactic acid), the so-called initial burst release was avoided and a release of platelet-derived growth factor-BB over 70 days could be achieved [52].

Even though several attempts to regenerate entheses have repeatedly been carried out in recent years, there is still no complete and functional solution to recreate their complexity in function and structure. The challenges of enthesis tissue engineering were also reviewed elsewhere, examples of recent review articles are [1,5,30,36,37,53]. As described above, the development of entheses during embryonic development is very extensive and influenced by many factors. Therefore it is difficult to imitate this development as a regenerative therapeutic approach. In addition, the different mechanical properties of the different zones must be precisely coordinated to ensure the necessary stability and tensile strength which goes along with unresolved biological questions. These issues mean that so far, a regeneration or replacement of an enthesis with all its components and properties was not successful which is resulting in an unmet clinical need.

## 3. Inflammation and the Immune System at Entheses

Considering the results of laboratories, preclinical and clinical studies, it becomes clear that the plethora of different approaches based on growth factors and stem cells, also called “regenerative therapies”, have still failed to show striking success in the clinic. This might be due to an inverse correlation between the regenerative capacity of organisms and their immune competence—the higher the latter, the lower the regenerative capacity [54]. In comparison to mammals, lower vertebrates can regenerate many of their tissues completely in a functional manner, instead of merely repairing them by the formation of scar tissue. Emerging from these findings, it is reasonable to postulate that novel biomaterials and release systems should be developed which are able to modulate the recipient’s immune system in order to potentiate the effect of stem cells and growth factors.

Here, we would like to advocate the idea that the impact of inflammation and immune reactions should be given greater consideration in future developments of regenerative therapies including biomaterials and implants. Anti-inflammatory and immunomodulatory therapeutic approaches could sustainably promote successful regeneration in many applications or only make it possible at all.

As already mentioned tissue damages or injuries are caused by infections, toxic and mechanical cues, degeneration or combinations of these factors. The body tries to heal such injuries by initiating immune responses and inflammations. During these processes, soluble inflammatory mediators bind to cell surface or cytoplasmic receptors. These receptors transmit signals into the cells by activating signaling cascades thereby leading to activation of target genes and ultimately to a secretion of additional soluble mediators. Immune cells, stem cells and tissue-resident cells are subsequently recruited by these mediators [55]. During the initial phase of inflammation, the innate immune system plays an important role while the adaptive immune system typically takes over in later stages. Overall, the immune system exerts both positive and negative effects on the success of the healing process. Ideally, an acute inflammatory reaction is followed by complete healing including functional restoration which is also known as regeneration. However, in mammals the regenerative abilities strongly depend on the state of development (fetal, neonatal, adult) [54]. Consequently, those events described in chapter 2 have not been recapitulated properly in animal models of human clinical questions to date.

Only a few years ago the scientific community realized that immune cells have a considerable presence in injured as well as in healthy entheses [56,57] although their numbers might be relatively low. The most important types of entheseal immune cells consist of interleukin 23 (IL-23) responsive cells like macrophages [58], natural killer (NK) cells [59] and γδ T cells [60]. The expression of IL-23 receptor (IL-23R)—as the major prerequisite of IL-23 responsiveness—on tendon, cartilage and bone cells within the enthesis remains to be determined. IL-23 is constitutively expressed in the human annulus in vivo and in vitro and is up-regulated in vitro by tumor necrosis factor-alpha (TNF-α) [61]. Macrophages derived from THP-1 (abbreviation for Tohoku hospital pediatrics-1) cells promote the osteogenic differentiation of mesenchymal stem cells through the IL-23/IL-23R/β-catenin pathway [62].

An inflammation at the enthesis is termed enthesitis and is highly important in the pathogenesis of spondyloarthropathies [12,56]. Spondyloarthropathies are a group of rheumatic diseases which are associated with inflammation at anatomically distal sites like tendon-bone attachments [63]. While IL-23 had already been suspected to be a part of the pathogenesis for some time [64], it has only been demonstrated a few years ago that it acts locally on entheseal resident IL-23R^+^ retinoic acid-related orphan receptor gamma T (ROR-γt)^+^CD3^+^Sca1^+^CD4^−^CD8^−^ T cells [63], which are similar to γδ T cells [65]. IL-23 binds to these receptors and might be of systemic origin. However, recent results suggest that CD14^+^ myeloid cells from the enthesis locally secrete IL-23 as was shown in vitro after a stimulation with mannan or lipopolysaccharide (LPS) plus interferon-gamma [56]. After binding of IL-23 the enthesis-resident T cells release inflammatory mediators like IL-6, IL-17, IL-22 and chemokine (C-X-C motif) ligand 1 [63,65,66]. Interestingly, the secretion of IL-22 leads to ectopic bone formation (osteophytes) and, in parallel, promotes osteoclast differentiation [67]. Furthermore, the secretion of IL-17A and TNF-α contributes to the bone loss observed in spondyloarthropathies. IL-17 also enhances neutrophil migration and activation. Neutrophils stimulate the inflammatory response by releasing proteases and reactive oxygen species which aggravate the pain associated with enthesitis [12]. As described here, IL-17 production has often been linked to upstream IL-23 signaling and termed IL-23/IL-17 Immune Axis (see as one example [68]) but recently it was shown that the production of IL-17 is IL-23-independent in γδ T cells in the gut [69].

The pathophysiology and treatment of enthesitis have been focal points of many studies and in 2017 an excellent review regarding these topics was published [12]. In order to contribute novel ideas we will focus on one specific signaling component which plays a central role in a multitude of signaling pathways but awaits to be investigated in normal and inflamed enthesis—TGF-β-activated kinase-1 (TAK1). This kinase may present an alternative for targeted therapy of enthesitis compared to the conventional use of nonspecific corticosteroids or non-steroidal anti-inflammatory drugs (NSAIDs). TAK1 is a downstream molecule in the IL-17-mediated signaling [70,71], suggesting a relevance of TAK1 in entheses. This theory is further supported by the already reported positive effect of the inhibition of its downstream target p38 (protein with molecular weight 38 kDa) during the repair of the rotator cuff [72]. Also in line with this hypothesis, the targeting of different protein kinases with a focus on kinases, which regulate the innate immune system has been discussed for the development of anti-inflammatory drugs and treatment of chronic diseases [73]. This may not only provide novel treatment options for chronic diseases with a direct manifestation at entheses such as spondyloarthropathies but also to additional chronic comorbidities such as metabolic diseases. As one example, it was demonstrated that reversal of prolonged obesity-associated cerebrovascular dysfunction could be achieved by inhibiting TAK1 in microglial cells (macrophages within brain [74]). Since comorbidities such as cardiovascular disease, obesity or diabetes mellitus may notably influence the course of disease and treatment response this stresses the need for novel treatment strategies in the musculoskeletal system and beyond [75,76]. Due to the lack of existing data for TAK1 in entheses, we will refer to studies in bone, cartilage and tendon and to the role of TAK1 in cells of the innate immune system exemplified by macrophages and on cells of the adaptive immune system exemplified by T cells, with the aim to shed some light on the potential role of TAK1 in these tissues, both under normal and inflamed conditions.

## 4. TAK1 (Transforming Growth Factor-β Activated Kinase 1)

TAK1, officially known as mitogen-activated protein kinase kinase kinase 7 (MAP3K7), is a ubiquitously expressed member of mitogen-activated protein kinases (MAPK) with unique functions within a number of signaling cascades. Initially, it was found that TAK1 is enzymatically activated by TGF-β which hence is its name-giving factor [77,78]. Among the mammalian TGF-β isoforms, the β_1_ variant was abundantly used to activate TAK1. Much less information is available for the β_2_ variant [79]. In case of TGF-β_3_, just an indirect evidence was obtained as the use of a TAK1-inhibitor inhibited its downstream signaling [80]. Furthermore, BMPs and other growth factors, like wingless proteins, granulocyte-macrophage colony-stimulating factor (GM-CSF) and macrophage colony-stimulating factor (M-CSF), induce receptor-mediated signaling cascades, which are able to use TAK1 as a signaling mediator protein [81,82,83,84,85]. Apart from its role in growth factor signaling during differentiation and regeneration, TAK1 is an important mediator in signaling of inflammation and in the overall immune response. Important activators of TAK1-dependent receptor-mediated signaling in inflammatory settings—such as spondyloarthropathies—include IL-1 [86], IL-17 [71], TNF-alpha [87] as well as other cytokines. Furthermore, toll-like-receptor (TLR) binding pathogens like bacteria or viruses and TLR binding agonists like LPS and poly I:C are also able to transduce their signals via TAK1 [88]. In the adaptive immune system, T [89] and B cell [88] receptors can facilitate TAK1 signals [88,90]. Despite this plethora of factors activating TAK1, neither IL-22- nor IL-23-mediated direct activation of TAK1 has been reported.

**Figure 2:**
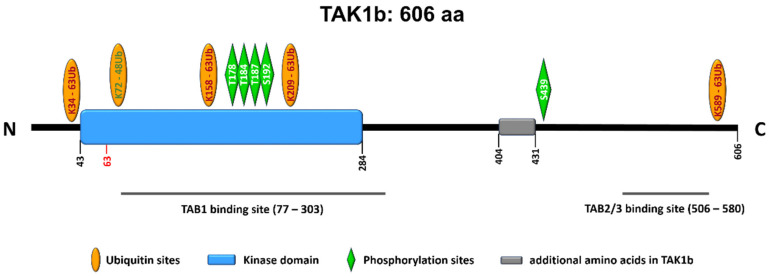
Linear sequence of TAK1b (606 amino acids). The second major splice variant, TAK1a, lacks the 27 amino acids between position 404 and 431. Important posttranslational modifications are indicated.

Subcellularly, TAK1 is mainly localized in the cytoplasm while also being able to translocate into the cell nucleus or associate with the plasma membrane. A constitutive nuclear localization of TAK1 has been reported in neutrophils for example [91]. The protein is transcribed in two major isoforms—TAK1a with 579 amino acids and TAK1b with 606 amino acids. The longer variant has 27 additional amino acids; otherwise the peptide sequences of both isoforms are identical, see Figure 2. For more information on the splice variants and their cell and tissue distribution, the reader is referred to publications by colleagues [92,93,94].

Signal transduction via TAK1 requires it to be initially activated via binding of lysine 63-linked polyubiquitin at lysines 34 and 158 [95,96]. For TAK1-dependent enzymatic activity and downstream signaling, the TAK1 binding proteins (TABs), TAB1, TAB2 and TAB3 are also important (Figure 2). Specifically, the formation of TAK1-TAB1-TAB2 or TAK1-TAB1-TAB3 complexes is required for the autophosphorylation-induced activation of TAK1 as reviewed by Hirata et al. [97]. Such autophosphorylation takes place at the threonines 178, 184 and 187 as well as serine 192 [97]. Another important phosphorylation site is serine 412 (in TAK1a) or serine 439 (in TAK1b) as depicted in Figure 2. This phosphorylation event is mediated by upstream kinases, for example, by protein kinase A [97]. Deletion of the 22 N-terminal amino acids also activates TAK1 kinase activity [77].

Site-directed mutagenesis of one of the four phosphorylation sites T178, T184 and T187 or S192, the mutation of lysine 63 into tryptophane (K63W) [77] or the mutation of lysine 158 into arginine (K158R) [95] lead to a loss of kinase activity.

Downstream signaling pathways activated by TAK1 include MAPKs such as extracellular signal-regulated kinases, p38, c-Jun N-terminal kinases (JNK), phosphatidylinositol 3-kinases (PI3K) and nuclear factor kappa-light-chain-enhancer of activated B cells (NF-κB) [98,99,100]. In conclusion, TAK1 is a highly important signaling protein which is present in all cell types and is activated by a vast array of cytokines including growth factors and chemokines, mitogens as well as cellular stress [97] and integrates different signaling pathways. In particular, differentiation and inflammation may be modulated and fine-tuned by TAK1 signaling.

For a better understanding of TAK1 functions, genetic systems have been used extensively. It has been shown that the constitutive knockout of TAK1 in mice leads to embryonic lethality, underlining the relevance of TAK1 for the normal development in mammalians [101,102]. Consequently, conditional knockout mice have been used to determine the role of TAK1 in vivo. Comparatively, little is known about the importance of TAK1 in the human system, including MSCs. In addition to genetic manipulations which provide stable or, if applicable, transient deletion or downregulation of TAK1 expression, the use of small molecule inhibitors which block the kinase activity of TAK1 represents another scientific approach. The inhibitors which are mainly used today provide permanent inactivation of TAK1’s kinase activity through a stable binding in the kinase domain. However, with continuous synthesis of new proteins by the cells and the degradation of inhibitor-bound TAK1, cells are able to resume TAK1 signaling.

### 4.1. Inhibitors of TAK1 Enzymatic Activity

Kinase inhibitors are divided into type I and type II. The decisive factor for this classification is the position of the asparagine-phenylalanine-glycine (DFG) motif at the N-terminus of the activation loop (amino acids 175–177 of TAK1). The DFG-motif can either be flipped “in” representing the active state of the kinase or “out” for the inactive state [103]. Up to now, the most commonly used type I inhibitor for TAK1 enzymatic activity is (5Z)-7-oxozeaenol [98], a small molecule which irreversibly binds to the cysteine 174 at the ATP-binding site of TAK1 [104]. Even though (5Z)-7-oxozeaenol shows promising results it has a notable amount of off-target effects on different kinases, which need to be considered in therapeutic applications. In order to optimize TAK1, inhibition by small molecules based on a 2,4-disubstituted pyrimidine scaffold were evaluated. These compound inhibitors function, analogous to (5Z)-7-oxozeaenol, via covalent binding to cysteine 174. The compound 5, as described by Tan et al. (2017), showed a similar potency to (5Z)-7-oxozeaenol while exerting a lower inhibition on other kinases [105]. Apart from this, different commercially available inhibitors like Takinib (type I) and NG-25 (type II) are used to target TAK1 activity. The latter is a potent TAK1 inhibitor but has an even wider range of off-targets than (5Z)-7-oxozeaenol [106]. Like the aforementioned compounds, Takinib was specifically designed to inhibit TAK1 and therefore exerts fewer off-target effects than (5Z)-7-oxozeaenol or NG-25. Interestingly, Takinib does not bind to cysteine 174 but rather slows down the auto-phosphorylation, which is required for TAK1 activation and later forms hydrophobic interactions and hydrogen bonds within the ATP-binding pocket, competitively inhibiting kinase activity [107]. Table 1 shows inhibitors, which are predominantly used in experimental studies in cell cultures and in experimental animals.

The application of TAK1 inhibitors in osteoarthritis might be especially attractive regarding the possibility of pain-relief in patients since TAK1 has been shown to be part of the increased nerve growth factor production in osteoarthritic synovium [108]. Another interesting approach to target TAK1 was based on a genetic approach in which the kinase was first inactivated and then reactivated to prevent heterotopic ossification in wound healing [109]. The authors also showed that a reduced TAK1 activity promoted the proliferation of tendon-derived cells, thereby limiting differentiation.

### 4.2. TAK1 in Musculoskeletal Tissues

In entheseal healing bone marrow stem cells can infiltrate into damaged areas where they are able to differentiate into osteo-, chondro- and tenocytes [110]. TAK1 seems to be part of these signaling pathways and acts via activation or inhibition of different downstream mediators. As mentioned previously, TAK1 has not yet been investigated in the entheseal context. Therefore, we will focus on its effects in bone, cartilage and tendon.

TAK1 exerts specific effects on bone formation. It is a major regulator of specific gene expression, like bone sialoprotein, osteocalcin and alkaline phosphatase, during osteogenic differentiation and its inhibition notably increases mineralization of MSCs in vitro. Additionally, an overexpression of TAK1 led to an impaired osteogenesis in MSCs [111,112]. The same effect could be observed after completely abolishing TAK1 using osteoblast-specific TAK1 knockout mice in vivo [111,113]. These findings showcase the complexity of TAK1-dependent signaling and could prove to be a challenge in clinical applications regarding bone healing. However, it has to be noted that the aforementioned studies inhibited TAK1 on the genetic level, meaning that no TAK1 was expressed for a prolonged time period. By using small molecules to inhibit the activity of TAK1, a far superior fine tuning of inhibitory effects should be possible. Since the protein is constantly degraded and reproduced, halting the application of inhibitors would most likely and quickly restore the initial rate of activity.

TAK1 is also connected to osteoblast survival. Apoptosis can be induced via dexamethasone and subsequently activates TAK1-mediated proline-rich tyrosine kinase-2/JNK signaling. Accordingly, inhibiting TAK1 results in restored osteoblastic cell survival [114]. In addition, for bone maintenance and remodeling another cell type is highly important—osteoclasts. Osteoclasts are derived from hematopoietic stem cells and strongly resemble monocytes or tissue resident macrophages. These multinuclear cells are responsible for bone resorption and can initiate immune responses making these cells mediators of bone maintenance, remodeling and repair. It has been shown that TAK1 plays a crucial role in osteoclastogenesis driving the maturation of monocytes into osteoclasts by activating NF-κB signaling. The inhibition of TAK1 in these cells results in loss of function and successive osteopetrosis or uncontrolled bone formation [115].

Beside its role in bone homeostasis, TAK1 was shown to be an important mediator in early cartilage development as its inhibition impairs BMP signaling, effectively reducing the formation of joints and cartilage in mouse embryos [116,117]. In contrast to these findings, in mature organisms an upregulated TAK1 activity is associated with osteoarthritis-like conditions mainly by disruption of cartilage homeostasis [118]. Under these conditions, the inhibition of TAK1 or its downstream mediators have proven to be viable methods to rescue chondrogenic differentiation in vivo [119] and in vitro [120].

A reduced TAK1 activity by use of the inhibitor (5Z)-7-oxozeaenol promoted the proliferation of tendon-derived cells, thereby limiting differentiation [109]. This observation fits well with the pivotal role of different TGF-β isoforms in driving tendon differentiation (chapter 2; TGF-β isoform not specified: [121], TGF-β_2_: [122], TGF-β_3_: [123]; a concise summary is found in Reference [124]). Additionally, TGF-β is needed to maintain tendon cell fate as it was shown that mouse tenocytes lose their differentiation markers following a conditional TGF-β type II receptor knockout [125]. Returning to an entheseal context, elevated TGF-β levels also revealed a tendency to promote tendon-to-bone healing in rabbits who received autologous MSC transplantations [126]. In contrast to members of the TGF-β superfamily, another important TAK1 activator, IL-1, exerts an undesirable influence on tenocytes. The proinflammatory cytokine induces the production of matrix metalloproteases (MMPs) and IL-6, leading to the digestion of extracellular matrix eventually diminishing biomechanical tendon properties [127]. A recent study suggests the use of embryonic stem cells as a possible solution. Due to their low IL-1 receptor type 1 and high IL-1 receptor antagonist protein expression, tenocytes derived from embryonic stem cells may prove to be a viable alternative for tendon or even entheseal regeneration [128]. These differences in the outcome of TAK1-mediated signaling show once more the possibilities of TAK1 as a target in tissue healing.

### 4.3. TAK1 in Immune Cells

We already pointed out that TAK1 is an important protein in differentiation, during inflammatory signaling and is able to mediate various cellular functions. In recent years, TAK1 has received more attention, especially within the context of immune cells. Macrophages are key mediators of these inflammatory processes. They are able to secrete a broad spectrum of pro- and anti-inflammatory cytokines as well as pro-inflammatory reactive oxygen species and MMPs.

TAK1 has been shown to play a pivotal role in macrophage development, essentially describing the central switch for the regulation of macrophage function and polarization [129]. In accordance with this, TAK1 has been used as a target in different pathologies such as cancer [107], tissue inflammation [130,131] and autoimmune diseases [132]. Since TAK1 promotes the pro-inflammatory M1 phenotype in macrophages [133], the aforementioned strategies of silencing or inhibiting TAK1 are used to alleviate inflammatory reactions. For example, the silencing of TAK1 proved to be a viable option to treat rheumatoid arthritis in mice joints [134,135]. It is further speculated that the inhibition of TAK1 induces a switch to the anti-inflammatory M2 phenotype in macrophages. This would be highly beneficial for entheseal wound healing as M2 macrophages are involved in tissue repair and homeostasis. Apart from its role in polarization TAK1 also functions as a safeguard against pyroptosis. Under TLR-stimulating conditions an impaired function of TAK1 results in the cleavage of caspase-8 and a subsequent pro-inflammatory cell death [136,137]. A recent publication points out differences between macrophage populations in their sensitivity to cell death by necroptosis induced by TAK1 inhibitors [138]. This highlights the extensive spectrum of TAK1 activity and demonstrates that using macrophage function as a target needs to be approached with caution.

γδ T cells, which express the γδ form of the T cell receptor (TCR), represent the minority of T cells in the blood in comparison to αβ T cells [139]. In contrast, in the enthesis, γδ T cells are a highly relevant cell type [60,63,140]. To date, the role of the enthesis-resident γδ T cells is not fully understood. However, it has been discussed that these cells might play an important role in bone metabolism and control tissue homeostasis at the tendon-to-bone attachment site [65]. Furthermore, it has been speculated that microdamage of the enthesis might activate the γδ T cells which in turn might activate tissue healing [65,141,142].

As described above, TAK1 is a critical mediator which is involved in a wide variety of cellular processes, including in T cell development [89]. The influence of TAK1 in γδ T cells in general and in enthesis-resident γδ T cells, in particular, has not been investigated yet. Therefore, we will focus on the effect of TAK1 in T lymphocytes in general as well as we are going to link the existing knowledge of the T cells to the enthesis-resident γδ TCR expressing subtype.

In T lymphocytes TAK1 is essential for the survival and homeostasis of peripheral T cells as well as for TCR-mediated MAPK activation and NF-κB signaling, which was shown by three independent studies [89,143,144]. Deletion of TAK1 in mature thymocytes suspended TCR-mediated NF-κB and JNK signaling and was linked to a higher sensibility to apoptosis [90]. However, in effector T cells TAK1 is not involved in NF-κB and JNK signaling, albeit being critical for p38 activation and cell proliferation [89]. In humans, T cell acute lymphoblastic leukemia is linked to TAK1 deletion, demonstrating that the expression of TAK1 is indispensable for T lymphoblasts [145].

TAK1 is not only a key player in T cell life cycle it also affects other adaptive immune system cell types like B cells. In B lymphocytes, TAK1 is critical for the antigen receptor signaling and B cell receptor (BCR)-mediated proliferation [88,143]. It has been shown that TAK1 is mandatory for the BCR-induced activation of NF-κB and JNK signaling [88,146], which in turn are critical for the innate and adaptive immune response [89].

Taking everything into account, TAK1 plays a pivotal role in the regulation of cell survival, proliferation and differentiation of immune cells in vertebrates and invertebrates [147].

## 5. Conclusions

Unfortunately, no complete and functional solution to recreate the complex function and structure of entheses is available to date, which results in an unmet clinical need. Biological factors might be a key to solve this challenge. However, the complex interaction of many signaling networks during embryogenesis, which build up a native enthesis, cannot be mimicked by regenerative strategies yet. We here propose that among many factors considered up to now, spatially and temporally controlled targeting of TAK1 as a node factor of inflammatory and regenerative signaling (including targeting of selected cell types) might contribute to improve medical solutions. Due to its broad range of action, the inhibition of TAK1 could be utilized to rescue different parts of the enthesis, namely, bone and cartilage. Additionally, inflammatory responses could be subdued by applying TAK1 inhibitors like Takinib or (5Z)-7-oxozeaenol. Nevertheless, it has to be noted that tendon to bone healing largely depends on unimpaired TGF-β signaling. This cell type-dependent variation of TAK1 function leads to an important challenge for possible clinical applications in the future. To ensure a successful treatment, potential drugs inhibiting TAK1 have to be administered in a cell-specific way. A first step could be a local application at the designated area of effect. However, it cannot be guaranteed that only a specific type of musculoskeletal cells would be affected by this treatment, which might in turn lead to a variety of unwanted adverse effects. Here targeting of macrophages could prove to be an interesting approach. These cells are known for their phagocytic properties and therefore exhibit a significantly faster uptake of vesicles than musculoskeletal cells. To successfully address such issues of a spatially or temporally controlled modulation of TAK1 activity this kinase needs to be further analyzed under precise entheseal viewpoints, maximizing the possibility for suitable treatments.

## Figures and Tables

**Table 1:** Frequently used TAK1 inhibitors in experimental studies. IC_50_-values depict the half maximal inhibitory concentrations and were determined using enzymatic assays as stated in the specific studies in References [104,105,106,107]. Structures were drawn with ChemDraw based on PubChem information.

Name	Structure	Mechanism of Inhibition	IC_50_ (nM)
**(5Z)-7-Oxo-** **zeaenol** (Type I)	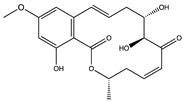	Binds covalently to the cysteine 174 of the active TAK1 with DFG-in formation of the activation loop. Thus, it permanently blocks the ATP-binding pocket.	9
**Takinib** (Type I)	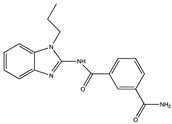	Slows down the auto-phosphorylation step in TAK1 activation. Competitively inhibits kinase activity via hydrogen bonds and hydrophobic interactions within the ATP-binding pocket.	9.5
**NG-25** (Type II)	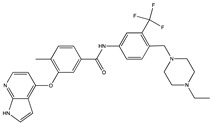	NG-25 binds to the ATP-binding pocket in the inactive DFG-out formation. It forms hydrogen bonds with different residues of the active site.	149
**Compound 5** (Type I)	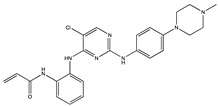	Binds covalently to the cysteine 174 of the active TAK1 with DFG-in formation of the activation loop. Thus, it permanently blocks the ATP-binding pocket.	50

## Abbreviations

BCR	B cell receptor
BMP	Bone morphogenetic protein
DFG	Asparagine-phenylalanine-glycine
GM-CSF	Granulocyte-macrophage colony-stimulating factor
HH	Hedgehog
IC_50_	Half maximal inhibitory concentration
IHH	Indian hedgehog
IL	Interleukin
*IL1RN*	Interleukin-1 antagonist (gene and cDNA)
IL-23R	Interleukin-23 receptor
JNK	c-Jun N-terminal kinase
LPS	Lipopolysaccharide
MAP3K7	Mitogen-activated protein kinase kinase kinase 7 (also known as TAK1)
MAPK	Mitogen-activated protein kinase
M-CSF	Macrophage colony-stimulating factor
MMP	Matrix metalloprotease
MSCs	Mesenchymal stem cells
NF-κB	Nuclear factor kappa-light-chain-enhancer of activated B cells
NK	Natural killer cells
NSAID	Non-steroidal anti-inflammatory drugs
p38	Protein with molecular weight 38 kDa
PI3K	Phosphatidylinositol 3-kinase
PTHrP	Parathyroid hormone-related protein
ROR-γt	Retinoic acid-related orphan receptor gamma T
RUNX-2	Runt-related transcription factor-2
SCX	Scleraxis
SOX9	Sex-determining region Y-box 9
TAB	TAK1 binding protein
TAK1	TGF-β-activated kinase-1
TCR	T cell receptor
TGF-β	Transforming growth factor-β
THP-1	Tohoku hospital pediatrics-1
TLR	Toll-like-receptor
TNF-α	Tumor necrosis factor-alpha
